# Foucault and medicine: challenging normative claims

**DOI:** 10.1007/s11019-023-10170-y

**Published:** 2023-09-25

**Authors:** Chris A. Suijker

**Affiliations:** 1 Department. of Urology, University of Groningen, University Medical Center Groningen, Groningen, the Netherlands; 2 Faculty of Philosophy, University of Groningen, Groningen, The Netherlands

**Keywords:** Foucault, Medicine, Objectification, Biopower, Medicalization, Normativity

## Abstract

Some of Michel Foucault’s work focusses on an archeological and genealogical analysis of certain aspects of the medical episteme, such as ‘*Madness and Civilization*’ (1964/2001), ‘*The Birth of the Clinic*’ (1973) and ‘*The History of Sexuality*’ (1978/2020a). These and other Foucauldian works have often been invoked to characterize, but also to normatively interpret mechanisms of the currently existing medical episteme. Writers conclude that processes of patient objectification, power, medicalization, observation and discipline are widespread in various areas where the medical specialty operates and that these aspects have certain normative implications for how our society operates or should operate. The Foucauldian concepts used to describe the medical episteme and the normative statements surrounding these concepts will be critically analyzed in this paper.

By using Foucault’s work and several of his interpreters, I will focus on the balance between processes of subjectification and objectification and the normative implications of these processes by relating Foucault’s work and the work of his interpreters to the current medical discipline. Additionally, by focusing on the discussion of death and biopower, the role of physicians in the negation and stigmatization of death is being discussed, mainly through the concept of biopower. Lastly, based on the discussion of panopticism in the medical discipline, this paper treats negative and positive forms power, and a focus will be laid upon forms of resistance against power. The discussed aspects will hopefully shed a different and critical light on the relationship between Foucault’s work and medicine, something that eventually can also be deduced from Foucault’s later work itself.

## Introduction

Michel Foucault (1926–1984) was a French philosopher whose main works focus on a historical analysis of social and institutional processes. His historical approach does not consist of an analysis of particular historical subjects, but the aim is to shed light on the systems of thought that ‘govern’ a particular period (Gutting and Oksala 2019). These systems of thought in Foucauldian analysis are named epistemes; the episteme of a particular era or period shapes the knowledge, conceptual possibilities and the discourse of that era or period, extending beyond the consciousness of any particular subject (Foucault 1976). This approach, often found in Foucault’s works, came to be the archaeological method in philosophical analysis. In order to account for transitions between several ‘archeological periods’, Foucault re-introduced the Nietzschean term ‘genealogy’, and Foucault mainly used genealogical methods to show that transitions in history were contingent, rather than rational and inevitable (Gutting and Oksala 2019).

Foucault was also concerned with the archeological and genealogical analysis of the medical discipline and sociomedical processes, trying to clarify the ‘episteme’ that characterized the generation of medical knowledge and discourse. In *Madness and Civilization* (1964/2001), Foucault investigates the structures of knowledge that governed discourse about madness and insanity. In *The Birth of the Clinic* (1973), Foucault studies the development of medical knowledge and the clinic, in which the medical clinic and the hospital adopted the ‘clinical method’ and came to be ‘teaching hospitals’ (Osborne 1998, p.31). Throughout his life, Foucault gave several lectures and lecture courses on the archeology and genealogy of (social) medicine and psychiatry (2000, 2003, 2004, 2008a, 2008b, 2016). In one of his last works, he focused on sexuality in *The History of Sexuality* (1978/2020a). His ideas about medicine do not remain limited to these works: one can find an elaboration on medicine’s relation to discipline and partitioning in *Discipline and Punish* (1977/2020b), a book that is mainly dedicated to the development of the penal system.

While Foucault was primarily occupied with providing archaeologies and genealogies of several aspects of the medical discipline, he seemed to shy away from making normative judgments about his analyses, as expressed clearly in his early work *‘Archaeology of knowledge’* (1976)^1^. However, other writers have studied his thoughts and filled in some of the ‘normative gaps’ that Foucault at first did not venture into (see footnote 1). For example, some of these writers tend to conclude, or argue, based on Foucault’s work, that certain processes are widespread in the medical discipline, and that these processes are unwarranted, unwanted and morally ‘bad’, which in turn partially confirms the ‘moral failure’ of the medical discipline (Peerson 1995; Svenaeus, in press; McDorman 2005; Adorno 2014). Processes often described in this light are those of objectification, power, medicalization, observation and discipline. These processes include various important concepts in Foucauldian thought that match Foucault’s attempt to grasp the ‘episteme’ of the generation of (medical) discipline, knowledge and discourse.

In this paper, I will provide a brief introduction and explanation of the main Foucauldian concepts that can be considered to be important, or are considered to be of importance by other authors, in describing the medical episteme. After introducing these main Foucauldian concepts, I will explore and critically evaluate several normative views put forward in the literature that appeal to these Foucauldian concepts. This normative dimension is very valuable to the way medicine operates, but as we know, Foucault himself remained quite implicit about the moral potential of his studies, focusing mainly on mechanisms of power and knowledge, except in his later studies (see footnote 1). Therefore, several writers have used his work to add a normative dimension to Foucault’s studies in describing the medical episteme. In this paper, I will primarily argue for the idea that the complexity of Foucauldian thought warrants both positive and negative moral evaluation. By doing so, I hope to add something to the critical discussion and dialogue surrounding the normative interpretation of Foucault’s work and the description of the medical episteme. In order to undertake this enquiry, I will appeal to Foucault’s own arguments and the arguments put forward by several other Foucauldian scholars.

Each section in this paper will focus on different Foucauldian concepts that can be considered to illuminate and normatively interpret the medical episteme. Section two will be concerned with the discussion of processes of subjectification and objectification, important concepts that are often invoked to describe processes of medical analyses and also related to Foucauldian thought. First, I will discuss the idea that doctors partake in processes of objectification, primarily through the clinical gaze that Foucault describes. I will discuss the contrast between this idea and possibilities for processes of subjectification and practices of the self. Section three dedicates itself to the debate on medicalization, mainly by focusing on the argument of the negation of death. Here, writers that endorse the view of death as unwarranted and negated by the medical discipline, based on Foucault’s concept of biopower, will be contrasted to other ideas of Foucault that describe death as forming the basis of life, knowledge and individuality. It can be seen that death remains an important concept in the description of the medical episteme. The last section, section four, will focus on panopticism (observation) and discipline, concepts very popular in Foucauldian thought, and also applicable to the medical episteme. In this section, I will further elaborate the relationships between the medical discipline, power and (room for) resistance, before drawing a conclusion to this study.

## Processes of objectification and subjectification

In one of his early works, *The Birth of the Clinic* (1973), Foucault discusses the clinical gaze (p.107–122). The clinical gaze is an important concept to the medical episteme and can be described as a kind of interaction, one that is not limited to seeing, between (several) doctor(s) and a patient (Foucault 1973, p.107–111). What is important to the clinical gaze is that it entails a certain epistemological baggage: knowledge, the gazing action, a linguistic construct, the tools through which perception takes place, and the perceived all work together in harmony. A linguistic and epistemological construct provide a focus and a boundary for the examination of the patient, else perceptual information would be infinite (Foucault 1973, p.111) and impossible to categorize.

An example is useful to highlight all these aspects of the clinical gaze. During a consultation with a patient, the physician might notice distended jugular veins. The physician combines what she sees with her medical knowledge. During this exact moment, it is clear that the clinical gaze combines observation with an epistemological construct. In our current ‘western’ epistemological construct, this will lead the physician to think that the patient might be suffering from heart failure. This in turn guides the subsequent exercise of her clinical gaze: with the use of a linguistic construct, she will ask questions to the patient that might lead to information and that might guide future inspections, such as touching the ankles for edema. Additionally, with her stethoscope, the physician will open up a new realm of visibility that contributes to the clinical gaze. Here it can be seen that the tool of the stethoscope, the questions asked through language, the knowledge of the doctor about illnesses, the visibility of the jugular veins and the touching of the ankles all combine rather harmoniously to visualize a certain pathology, and therefore, these actions all contribute to the clinical gaze.

Several writers have introduced the idea that Foucault’s clinical gaze is a process of objectification and (therefore) harmful to the patient. Anita Peerson states that through the clinical gaze, one localizes and configurates the disease, which contributes to processes of objectification (1995, p.108). Based on the role of the clinical gaze in quantitative and qualitative measurements, she eventually concludes: “What medicine ignores is the patient’s subjectivity” (Peerson 1995, p.108). In likewise fashion, Frederik Svenaeus also appeals to Foucault’s clinical gaze in order to describe the way in which the body becomes a foreign territory to the patient, including dehumanization through medical technology (in press). He directly equates these aspects of the clinical gaze with processes of objectification (Svenaeus, in press). Additionally, N.D. Jewson concludes, while using the concept of the clinical gaze, that “the sick-man became a collection of synchronized organs” (1976, p.229).

However, based on a closer reading of Foucault and some of his scholars, I would argue that these writers are only partially justified in drawing these conclusions. For me, Foucauldian thought seems to embrace both processes of objectification and subjectification in describing the medical episteme, something that Thomas Osborne also points out. He defends that the clinical gaze does not solely partake in objectifying processes, but that it also affirms the patient’s subjectivity (1992; 1998). Osborne partly bases his argument on the following passage, found in *The Birth of the Clinic*: “The gaze is no longer reductive, it is, rather, that which establishes the individual in his irreducible quality. […] The object of discourse may equally well be a subject, without the figures of objectivity being in any way altered” (Foucault 1973, p. xiv). With the clinical gaze, which also consists of a linguistic construct, physicians are able to start a dialogue, show interest and will make personal contact with the patient, which results in the idea that medically gazing is not like gazing at objects. The complexity of the medical gaze shows that, at least for Foucault, it is not necessary to establish, or rather impossible to establish whether a subject or an object partakes in the clinical encounter, and it can also be shown that in the clinical encounter neither objective nor subjective components are left out (Osborne 1992, p.84–85).

Additionally, Osborne argues that observing the individual and pointing out pathologies is not necessarily a process of objectification (Osborne 1992, p.83). It is through observation and localization that the individual appears at all in a Foucauldian sense (Osborne 1992, p.84), and it can also be seen that a disease is not an objective entity that is able to influence the structures of a person’s body, but that disease becomes a phenomenon of the organism itself (Osborne 1998, p.39–40). The disease has an “individual figure” (Foucault 1973, p.168–169): each disease has a different form and embeds itself in the patient (rather than invading the patient), partially affirming the individuality of that particular patient. This makes localization of disease rather a process of subjectification: it contributes to the establishment of a certain uniqueness of the particular organism in question. This can also be seen in patient encounters in the hospital: many patients eventually feel that the disease ‘has become part of themselves’, invading every aspect of their lives (Suijker et al. 2021).

Lastly, one can see that the clinical gaze also depends on the doctor, who performs subjective perception herself (Osborne 1998, p.40–41). In Foucault’s work, one reads: “everything or nearly everything in medicine is dependent upon a glance or a happy instinct, certainties are to be found in the sensations of the artist himself rather than in the principles of the art” (Foucault 1973, p.121). This form of perception, that aids processes of subjectification in the doctor-patient encounter, merits an interpretation as an aesthetic type of perception whose acquirement cannot be solely from books and theory: one learns it at the bedside of the individual patient (Foucault 1973, p.120–121; Osborne 1992, p.85; Osborne 1998, p.32). Subsequently, this type of ‘personal and artistic’ perception of the doctor is aided by certain medical instruments or technologies, such as the stethoscope (Osborne 1998, p.35), which do not necessarily make these instruments a sole tool for processes of objectification, even though their purpose at first seems to be to establish objective parameters and collect data. By following Foucault’s later line of thoughts, one can even argue that physicians construct a portion of their ethical identity on the basis of this kind of aesthetic and personal perception directed at patients (Foucault 1985; Scott 1992).

To elaborate on these points, based on my experience in medical practice, and on conversations with patients and other physicians, the skillful physician particularly creates a keen balance between subjectivity and objectivity while ‘clinically gazing’. In the example about suspected heart failure posited above, ‘objective parameters’ such as auscultation with a stethoscope are important, but the physician also focusses on the subject. The clinical gaze uses a linguistic construct and might posit questions such as ‘Do you like to walk and how far are you able to walk without getting short of breath?’ and ‘What kind of work do you normally do and is this still manageable?’. These questions, of course leading to medical knowledge and generating answers that might point to certain pathology, also touch on the subjective side of the patient. Simply put: what she/he normally likes to do. Therefore, the medical gaze seems to combine objective parameters with some very subjective ones. In this light, information that initially points towards pathology can also help with the subjective management of certain disease after diagnosis, such as focusing on rediscovering the ability to walk comfortably in the patient who liked walking. I think this last idea corresponds nicely to Foucault’s idea of the embedded “individual figure” (1973, p.168–169) of disease discussed earlier.

It could very well be that the writers who concluded that Foucault’s clinical gaze is primarily a process of objectification that leaves out subjective aspects failed to notice some of the rich aspects pointing towards subjectivity in Foucault’s work related to the medical episteme because of the fact that other philosophical works also speak about objectifying or reductive ‘gazes’. A possible explanation could be based on phenomenological strands of thought, in which Merleau-Ponty’s and Sartre’s ideas of the objectifying ‘look’ or ‘gaze’ (Merleau-Ponty, 1962/2012, p.366–378; Sartre, 1957/2003, p.276–326) have gained widespread attention in philosophical thought. This might have overshadowed a proper meaning of the concept of the clinical gaze explained above in Foucault’s often-ignored work *The Birth of the Clinic* (Osborne 1992, p.63). However, it could also be that Foucault’s earlier work conflates with his later thoughts, since his *Discipline and Punish* (1977/2020b) and his concept of biopower are also often associated with processes of objectification.

Continuing this strand of thought, David Armstrong equates the patient with a prisoner who is subject to a surveillance and discipline apparatus (which will be merited a more thorough analysis in section four). Armstrong concludes that “The prisoner in the Panopticon and the patient at the end of the stethoscope both remain silent as the techniques of surveillance sweep over them” (1987, p.70). Additionally, in describing examinations, Hubert L. Dreyfus and Paul Rabinow quote Foucault in stating that the examination “manifests the subjection of those who are perceived as objects and the objectification of those who are subjected” (Foucault, 1977/2020b, p.184–185). Of course, examinations are important and play a central role in the medical discipline (Dreyfus and Rabinow 1982, p.158–159). Eventually, through surveillance, documentation, visibility and examination, the modern individual is subject to procedures of objectification by the medical discipline (Dreyfus and Rabinow 1982, p.158–167). Therefore, at this point, Foucault’s earlier work reunites itself with aspects of subjectivity as the arguments above try to show, but this venture might not be fruitful for his later pieces of work.

Nevertheless, by concluding that Foucault’s later works prove that the medical discipline is thoroughly participating in objectifying practices also misconceives Foucault’s writings. Simply put, the medical episteme does not solely consist of the objectifying medical examination, but it also entails friendly conversation and discussion of a patient’s problems and thoughts. This must be something Foucault considered as well, since Dreyfus and Rabinow point out that in Foucault’s works after *Discipline and Punish*, such as *The History of Sexuality* (1978/2020a), Foucault primarily focusses on processes of subjectification once again. In these works, the careful reader can conclude that the medical episteme partakes in confessional technology, which entails the idea that a patient confesses about her problems, thoughts, desires, illnesses and troubles (Foucault, 1978/2020a, p.59), focusing on practices of the self (Foucault 1985). This leads to processes of subjectification: the ‘confessions’ have to be interpreted by the medical discipline and its content is thoroughly subjective (Dreyfus and Rabinow 1982, p.173–183), something that especially can be seen in the office of the general practitioner, who even frequently visits patients at home, but also in the offices of other specialists.

In the end, one might conclude that “practices of our culture have produced both objectification and subjectification” (Dreyfus and Rabinow 1982, p.203), an idea that is also consistent with Foucault’s own thoughts (Foucault 1982; 1985). The conclusion that Foucault’s ideas of the medical episteme mainly consists of processes of objectification therefore seems unwarranted for both his early and his later works. When interpreting Foucault, it seems more in place to adhere to a rather complex and symbiotic view that consists of a continuous intertwinement of patients, physicians, subjective and objective parameters all in order to superficialize disease and to enlighten us about the importance of well-being for the subject, and not to dehumanize people or ignore subjectivity as some writers claim (Peerson 1995; Svenaeus, in press).

## Death and biopower

Occasionally, writers have used Foucault’s work in order to depict the relationship between death and the medical episteme. This portrayal often invokes the concept of biopower, a concept central to Foucauldian thought and considered to be very important in the description of the medical episteme. Biopower is a form of power, often associated with economic or political incentives, that closely relates itself to the management and control of the living subject: it “exerts a positive influence on life that endeavors to administer, optimize, and multiply it, subjecting it to precise controls and comprehensive regulations” (1978/2020a). In order to exercise power over the individual, biopower uses mechanisms such as healthcare, statistics, urban planning and methods of normalization. Eventually, through all these mechanisms, a high degree of control and discipline is instantiated and power structures eventually take charge of the bodies and lives of the population, managing it at the will of the institutions and mechanisms that bring about biopower (Gutting and Oksala 2019).

In this manner, according to biopower, there is no room for death in the medical episteme, since death is the exact opposite of what the processes of biopower are trying to achieve: total control over life itself (Peerson 1995, p.109). Therefore, biopower, in avoiding death, leads to the promotion of ‘the healthy life’ and the avoidance of illness and other risks, reinforced by health policies, but also by social structures and the medical discipline. As Peerson argues, the medical episteme is characterized by the idea that one should take care of herself, and if this fails and results in illness, one is “morally obliged to seek a return to health” (1995, p.109). On the other hand, Peerson concludes that biopower strengthens views such as that death can be seen as a moral failure, that countries can be blamed for their mortality rate, and that the relatives of the deceased person blame themselves for “not having done enough” to prevent death (1995, p.109), since death is something to be avoided following the principles of biopower. Doctors are also in awe, since the discipline does not advance itself enough to prevent death and because doctors cannot always fulfil their Hippocratic Oath, failing total execution of principles of biopower.

Todd F. McDorman argues, based on Foucault’s writings, that biopower affects the right-to-die of the individual, and that the state, rather than the individual itself, is able to decide when it is time for a person to die or not (McDorman 2005). This is relevant to the medical discipline, since McDorman introduces the idea that the medical discipline focuses on mastering the body and life though medical technologies, such as “life support systems, feeding tubes, and respirators” (2005, p.266). Furthermore, the physician is seen as the instrument of transmitting the ideology of biopower, stigmatizing death (McDorman 2005, p.265–268). Death and the dying are a nuisance, and the ‘theatre’ of death has become the hospital, an unpersonal and foreign surrounding, eliminating aesthetics that previously surrounded death.

Francesco Adorno shares the views of Peerson and McDorman, concluding, “the dying man from now on occupies an asocial position that enables the repression of death” (2014, p.106). In addition, “funerals, practices of mourning, and rites pertaining to the dead have not entirely disappeared, but their lack of meaning is more and more evident” (2014, p.106). In the end, the biomedical discipline is primarily responsible for this negation of death (2014, p.111). Additionally, for Adorno, the role of suicide-prevention in the medical episteme is also something that biopower takes very seriously, since suicide is the excellent form of resistance against biopower (2014, p.108–110). The principle of biopower is life, and the goal of suicide is to negate this supreme principle. The danger of suicide to biopower led, according to Adorno, to a focus of the sociological (and medical) discipline on suicide, including therapeutic interventions and prevention (2014, p.108–110).

To sum these views up, the medical discipline withholds the individual from its ability to die when she/he wants and how she/he wants, by preventing death through several ‘programs’. First, life support systems are developed and applied, which prolongs life. Second, suicide prevention programs are in place to prevent people from performing ‘ultimate resistance’ against Biopower. Lastly, the medical discipline stigmatizes death by convincing populations, stripping away rituals surrounding death and using statistics to compare death rates.

Trying to summarize these points, McDorman quotes Foucault in saying that “Behind the doctor’s back, death remained the great dark threat in which his knowledge and skill were abolished” (1973, p.146; McDorman, p.2005). However, contrarily, I am of the opinion that based on Foucault’s work, one can also conclude that the role of doctors and the medical episteme in the negation of death is less than these writers argue. This does not imply that the view that biopower leads to a denial of death is false, since the arguments provided by the writers mentioned above do contain intriguing truths warranting critical evaluation, but merely that the role of the doctor and the medical discipline in these processes of biopower have been overestimated. This is because from another point of view, doctors and the medical episteme also seem to ‘embrace’ death instead of denying it, a thought that seems to have received less attention when considering Foucault’s work.

Foucault describes in his *Birth of the Clinic* that, even though a doctor’s knowledge and skill were first abolished in the face of death, eventually medicine was freed from the fear of death (1973, p.146). In the beginning of the 19th century, primarily due to the practices of Bichat, opening up corpses to study pathological anatomy regained popularity (Foucault, p.124–146). In the corpse, one can study the several tissues and forms of pathology. This eventually led to theories that were able to match alterations in tissues and sets of symptoms in the living. Foucault states that “the medical gaze must therefore travel […] vertically from the symptomatic surface to the tissual surface, in depth, plunging from the manifest to the hidden” (1973, p.135).

Eventually Foucault concludes, “life, disease and death […] form a technical and conceptual trinity” (1973, p.144). Disease throws itself into the bond between life and death, since a disease is not an acquired process anymore, but an internal process, gradually changing life into death (Foucault 1973, p.155). Additionally, death becomes a locus for epistemological value: pathology slowly destructs life, and pathology can be studied in corpses. Foucault concludes that for Bichat, “knowledge of life finds its origin in the destruction of life and in its extreme opposite; it is at death that disease and life speak their truth” (Foucault 1973, p.145). Eventually, “death left its old tragic heaven and became the lyrical core of man: his invisible truth, his visible secret” (Foucault 1973, p.172).

After reading the passages of death in *The Birth of the Clinic*, concluding that McDorman pulled the quotation of Foucault on death posited above out of its original context is not farfetched. Thomas Osborne additionally concludes, in the same light as the quote introduced in the previous paragraph, that death is constitutive of the individual, since the study of anatomical pathology in corpses caused each individual to be a legitimate object of knowledge (Osborne 1992, p.73–74; 1998, p.36–37; Foucault 1973, p.170). Death, in Foucault’s treatment of the medical episteme, therefore does not seem to be completely negated by the medical discipline, as Osborne also points out (1992, p.72–75).

As Foucault merely discusses the movements in the 18th and the 19th century in *The Birth of the Clinic*, authors could argue that the position of the medical discipline on death has changed. I venture to state that death still occupies a central place in the education and professional practice of doctors. Dissection and demonstration of cadavers remains to be an important tool in medical education in almost all universities (Elizondo-Omana, Guzman‐Lopez & De Los Angeles Garcia‐Rodriguez, 2005), including my affiliated university. Additionally, one sees in the widespread pathological study of tissue from passed individuals that the medical discipline shows it is actively preoccupied with (material of) corpses. Lastly, implicitly connected to Foucault’s thoughts, is the role of the medical discipline in organ procurement. While on the one hand probably contributing to biopower by the relocation of organs, organ procurement on the other hand demonstrates that doctors do not enforce the stigmatization, negation and amoral character of death, since organ donors might be ‘praised’ for dying (Prottas and Batten 1988; Childress 2001).

Of course, almost all procedures surrounding death in the medical discipline have the aim to deliver a better life, which is consistent with the concept of biopower, but I think that the ideas discussed above weakens the views that the medical episteme negates and stigmatizes death, besides enforcing the idea that death is a moral failure as has been claimed by several authors. In addition, asking patients openly about their treatment restrictions concerning resuscitation, intubation, mechanical ventilation and intensive care admission is routine nowadays. Secondly, in some countries, doctors, especially general practitioners, created a sphere in which dying is even facilitated through palliative sedation and euthanasia. In the Netherlands during 2017, 6460 people died by active euthanasia, which amounts 4.33% of all deaths during that year (Groenewoud et al. 2021), not even counting passive palliative sedation. Euthanasia is being performed by the general practitioner in 87% of cases, and in the Netherlands, general practitioners can even specialize for an additional 1.5-2 years in end-of-life and palliative care.

It therefore seems that although the writers discussed above are right in contending that medical discipline is a field of practice which generally promotes ‘life’ and tries to postphone death through several means, care is to be taken in interpreting the role and attitude of the medical discipline and doctors in the matter. The argument that most physicians and the medical discipline as a whole are actively ‘negating and stigmatizing’ death seems to merit nuance, since doctors and the medical discipline have dedicated themselves to studying ‘death’ as a basis for knowledge, and since they also promote a ‘ethical’ and personal choice for death, subtle aspects of the discussion that can be partially found in Foucault’s own works.

## Panopticism and resistance

Leaving discussions on processes of objectification, subjectification, biopower and death aside, this section focusses on the panoptic apparatus, its applicability to the medical episteme and the ethical connotations writers often attach to the idea of panopticism in the medical discipline. Jeremy Bentham first introduced the idea of a panoptic institution, but in philosophical discourse, Michel Foucault popularized the concept. A panopticon is a round structure, with a ‘guard tower’ in the middle of the building, and ‘cells’ at the periphery (Foucault, 1977/2020b, p.195–228). From the guard tower, it is possible to watch what happens in every cell, and if one blinds the windows of the guard tower, the inmates have no idea at what moment they are being watched or not, leading to the fact that behavior can be controlled or power is exerted by the idea that someone might be watching, even though no-one is in effect surveilling the inmates. This leads to the idea that inmates eventually internalize the idea of being the subject of surveillance at any time, resulting in constant exhibition of ‘normal behavior’. Panopticism has inevitable connections with Biopower, since surveilling people makes it easier to subsequently discipline the body and ‘life’ itself. Linkage to the partitioning of space is also apparent, since division of space into individual units is necessary in order for Panopticism to work.

Michel Foucault further extended this concept to societal structures, applying it to the study of different types of normalizing behavior. In a medical example, the guards could signify health experts or family members that observe a person’s health and personal development. The individual surveilled eventually internalizes the ideas for which she is the subject of observation, partly because she does not know when one observes her or not. She comes to acquire goals that conform to ‘normal’ behavior; “she becomes her own guardian” (Dreyfus and Rabinow 1982, p.189). Eventually, she will practice sports, eat healthy and dress normally, since else, scrutinization will occur or she will scrutinize herself. This example also highlights the mutual arrangement between panopticism and biopower.

In *Discipline and Punish*, Foucault further clarifies the relationship between medicine and discipline/panopticism: “Gradually, an administrative and political space was articulated upon a therapeutic space; it tended to individualize bodies, diseases, symptoms, lives and death […] Out of discipline, a medically useful space was born” (1977/2020b, p.144). In a later passage, Foucault introduces the idea of medical disciplinary power and observation that extends from the hospital to the population (1977/2020b, p.212). Anita Peerson eventually concludes, based on Foucault’s ideas, that “With all the data before it, medicine became all powerful and set the agenda for citizens to conduct physically and morally healthy lives” (1995, p. 111). Data here refers to documenting statuses of illness, health, birth, and death, both on an individual and a population-wide level.

Eventually, the medical discipline also became concerned with urban planning and controlling epidemics, since environmental factors could drastically alter conditions of health and disease (Foucault 2000). David-Olivier Gougelet infers that “The various authorities concerned with public hygiene and the population’s medical practices – and, ultimately, the mechanisms of biopower – succeeded in establishing and subsequently exerting their control over that environment and its inhabitants […] in the name of their well-being” (2010, p.61). In this passage, the reader is also able to identify the parallel between panopticism and biopower.

While not explicitly discussing panopticism himself, Alan Peterson concludes, based on Robert Castel’s work, that the observation of individuals and population has shifted to analyzing factors of risk (Peterson, 1997, p.345–349). Nettleton (1997) also discusses the importance of risk in biopower, and implicitly to panopticism. Factors of risk deliver a widespread array of possible fields for preventive intervention. Of course, the medical discipline plays a major role in calculating risks, primarily through epidemiological endeavors, which has become a prime field of study in the medical episteme (Peterson, 1997, p.353). Eventually, Peterson argues for the idea that “everything potentially is a source of ‘risk’ and everyone can be seen to be ‘at risk’” (1997, p.350). The medical discipline also plays a role in enforcing risk-avoidance behavior by giving ‘expert opinions’ (Petersen 1997, p.346). Lastly, it is generally thought (and taught) that individuals exercise control over their own body, which makes the evasion of risks a personal responsibility (Petersen 1997, p.354–359; Nettleton 1997).

In these analyses, it is pictured that power exercised by these mechanisms seems to all-pervading and all-powerful. It is paramount to notice that Foucault and his scholars generally agree upon the idea the medical discipline is not confined to these power mechanisms: all kinds of institutions and microprocesses exercise the mechanisms and the medical discipline is not the sole ‘malefactor’. Often Foucault’s concept of governmentality envelops all these aspects, a concept that describes and entails the techniques of power and the conduct of the population (Gutting and Oksala 2019). In the discussion of health and governmentality, the medical discipline is part of the whole process, and the medical episteme can be seen to exhibit the same kind of processes and power mechanisms (Osborne 1997; Petersen 1997; Nettleton 1997; Gougelet, 2010).

But what is also important to notice in the discussion related to discipline, power and the medical episteme, is that power also merits positive interpretation. Deborah Lupton points to the idea that Foucault stresses the productive aspects of power (1997): it can be seen that power “doesn’t only weigh on us as a force that says no, but that it traverses and produces things, it induces pleasure, forms knowledge, produces discourse” (Foucault 1984, p.60), focusing on Foucault’s opinion that sometimes power structures enable us to perform practices of the self (Foucault 1985). Medicine, as Lupton argues for, is a field that shapes its own objects and discourses, and therefore, the doctor bring the phenomena of his patient and his illness into being (1997, p.195–196). This power is not exercised through coercion, but rather through persuasion, which makes doctors, according to Lupton, “links in a set of power relations” (1997, p.196). This particularly coincides well with the feeling that doctors have when performing a consult with patients. Doctors know they might hold a certain power over patients and their health, but they try to exercise the positive aspects of this power by trying to lay bare illness and treat it for the welfare of their patient, not because the doctors feel they are part of a construct of governmentality. Doctors perform these consults as a deliberative and informative dialogue instead of a coercive monologue, just as medical perception can be seen as an ‘aesthetic’ type of perception performed by ‘artists’ as discussed above in section two.

Another interesting aspect is that Lupton also stresses the idea of resistance in these power relations. She mentions that Foucault highlights that “where there is power there are always resistances, for power inevitably creates and works through resistance” (Lupton, 1997, p.200). One can observe such (micro-) resistances against medical power empirically during patient-doctor consultations. During these consultations, patients might respond by “direct rejection […], non-cooperation, silence, escape, avoidance, and […] concealment” (Lupton, 1997, p.203). Compliant patients are patients who are “engaging in practices of the self that they consider are vital to their own well-being and freedom from discomfort and pain” (Lupton, 1997, p.205). The fact that a lot of patients seem to completely avoid doctor consultations with certain problems affirms this idea, which results in the idea that for doctors, incidences of certain ‘diseases’ in medical offices are not at all adequate reflections of the incidence in the general population (Suijker et al. 2021). A subject still seems to decide that his problem is worth consulting a doctor for, and the subject probably does that based on her own preferences, and therefore, patients also seem to have an important role in the medical episteme. One could however still hold the idea that the individual ideas of vitality, which form the basis for resistance and avoidance and the object of the ‘persuasion of the physician’, are unmistakably internalized through societal processes, a component of Panopticism. This aspect is however only partially the result of mechanisms of the medical discipline, and seldom the result of enforcement by the medical discipline.

Lastly, doctors sometimes exercise resistance against biopower itself. In the very elderly or the very sick patient, where chances of regaining good quality of life are scarce, doctors sometimes adopt a laissez-faire stance, in which they present information to patients in a way in which doing nothing or palliative sedation might turn out to be the best option, even though technically other options might improve survival. For example, the doctor might frame a very elderly patient’s thoughts about imaging and investigation of suspected bowel cancer by highlighting the negative aspects of such additional investigations (e.g. hospital admissions, transportation time, chance of complications). In turn, the doctor might suggest that additional medical examination is not be a wise step, upon which the patient might more easily agree than when the patient was solely told that additional investigation might confirm ‘malicious disease’.

Eventually, the whole discussion on how to interpret Foucault and his concepts used to describe the medical episteme might boil down towards ethics. On the one hand, there is the ethics of humanitarian reason: medicine, through reason, cures disease, fights epidemics and health triumphs over sickness (Rose 1998, p.66–69). On the other hand, there is the ethics of accepting and embracing bodily suffering. Nikolas Rose notices that the former, humanitarian ethics, received the upper hand in contemporary society. This led to medicine extending beyond illness, into every aspect of care and “into the management of normality itself” (Rose 1998, p.67). Medicine is the expert of life itself; it leads to a postponement or an annihilation of (metaphysical) suffering, it instantiates an ethic of happiness, and therefore, “medical thought is fully engaged in the philosophical status of man” (Foucault 1973, p.198). Ultimately, Rose concludes that “for a historian of the present, to recognize this is not to condemn it” (1997, p.69), a quote that I believe perfectly fits Foucault’s thought and this study.

## Conclusion

The aim of this paper was to explore the relation between Michel Foucault’s work and the medical discipline. In each section, different aspects of Foucault’s thoughts and its consequences for medicine has been discussed. As can be seen, Foucault’s concepts have been invoked by several writers to discuss and indicate developments and processes of the medical episteme, often with a normative interpretation or connotation. The subtlety of Foucault’s work and his seemingly varying stance on such matters during his career as a writer makes it difficult to draw clear conclusions or to normatively interpret his work, as can also be seen while reading this article.

By analyzing Foucault’s work thoroughly, and that of his scholars, it seems natural that the concepts that he invokes and the way he analyses processes warrant a kind of normative interpretation since his thought is so valuable and is essentially rich in implications for societal matters. Several writers have ventured quite successfully into this enquiry, but this article shows that it is difficult to draw clear normative conclusions considering Foucault’s work. It can be seen that writers have discussed the role of objectification, biopower, the negation of death and panopticism in the medical episteme and that these writers have quite courageously asked the question whether these processes are to be valued or not. However, it can be seen that when Foucauldian thought is reconsidered, other conclusions can be drawn as well: there seems to be room for subjectivity, practices of the self, embracement of death, positive forms of power, and resistance against forms of power, which gives way to a kind of ambiguous atmosphere when applying Foucault’s work to practical and/or normative matters.

This highlights the importance of the need to be careful when performing a normative reading of Foucault. His thought is a rich and complex interrelation of concepts such as objectivity, subjectivity, knowledge, negative and positive forms of power, the body and the self, and I think that the processes he describes have both negative and positive normative aspects, partially also depending on the situation one encounters herself in. The most important thing when considering Foucault’s work in relation to the medical discipline is the accurate and valuable description that Foucault’s offers through his important concepts in order to illuminate the workings of the medical episteme. What remains to be an important quest for Foucauldian scholars is to continue critically evaluating and reflecting on his work, optimizing the dialogue surrounding the abundant possible normative consequences and implications of these cherished philosophical works, especially considering ever-changing practices of knowledge and power such as the medical episteme.

## Data Availability

Not applicable.

## Footnotes

1 However, it is of importance to note that in his later work, such as *‘The History of Sexuality’* (1985) Foucault interestingly seems to partially abandon this perspective, performing normative analyses by focusing on the morality of behaviors and practices of the self. How much room there is for normative thought in Foucault’s own works is a slightly different question compared to how interpreters of Foucault relate his work to the medical discipline from a normative perspective, which has been the aim of this study. Nevertheless, this point will be stressed in the article when it is seen productive for the arguments made, but an extensive future study focusing on normative claims or analyses solely in Foucault’s own works might do this particular subject more justice.
